# Symptom burden and its effect on the quality of life in a selected group of breast cancer patients in Delta State (Nigeria) after chemotherapy: a cross-sectional study

**DOI:** 10.11604/pamj.2026.53.7.46948

**Published:** 2026-01-08

**Authors:** Deliverance Brotobor, Chinomso Nwozichi, Osahogie Isaac Edeawe, Onoriode Brotobor

**Affiliations:** 1 Department of Nursing Science, Delta State University, Abraka, Delta State, Nigeria,; 2 Kennesaw State University, Georgia, United States of America,; 3 Department of Pharmacology and Therapeutics, Ambrose Alli University, Edo State, Nigeria,; 4 Department of Surgery, Federal Medical Centre, Asaba, Delta State, Nigeria

**Keywords:** Breast cancer, clusters, chemotherapy, symptom burden, fatigue, pain

## Abstract

**Introduction:**

breast cancer remains a significant global health burden, with chemotherapy serving as a cornerstone of treatment. However, chemotherapy is associated with a high symptom burden, adversely impacting patients' quality of life. This study assessed the prevalence, severity, and impact of symptom burden among breast cancer patients undergoing chemotherapy in Delta State, Nigeria.

**Methods:**

a cross-sectional design was utilized, and data were analyzed using Chi-square tests to examine associations between symptom burden and sociodemographic factors, cancer stage, and overall well-being. The researchers purposively recruited 139 participants of the 195 patients from the Oncology Clinics of Delta State University Teaching Hospital, Oghara, and Federal Medical Centre, Asaba, both in Delta State, Nigeria.

**Results:**

findings revealed that 36% of patients experienced moderate symptom burden, while 35.3% had severe or very severe burden. Pain (80%), fatigue (65%), and sleep disturbances (60%) were the most common symptoms. Marital status (χ^2^= 19.674, p = 0.034), duration since diagnosis (χ^2^= 11.149, p = 0.047), and cancer stage (χ^2^= 8.292, p = 0.007) were significantly associated with symptom burden, while age, education, and employment status were not. A strong inverse correlation was observed between symptom burden and quality of life (χ^2^= 19.828, p= 0.0225), with increasing symptom severity leading to diminished physical, emotional, and social well-being. Patients with no symptom burden reported the highest quality of life, while those with severe and very severe burden experienced the lowest well-being.

**Conclusion:**

this study underscores the importance of individualized supportive care tailored to symptom profiles, particularly for patients with advanced-stage disease and those lacking social support. Addressing symptom burden holistically can improve treatment adherence, resilience, and overall well-being in breast cancer survivors.

## Introduction

Breast cancer is one of the most prevalent and invasive malignancies globally, with incidence rates continuing to rise [1,2]. According to the World Health Organization, approximately 2.3 million new cases of women were diagnosed, with breast cancer in 2020, leading to 685,000 deaths annually [3]. While breast cancer can affect individuals of all ages, its prevalence is higher in older women [4]. The high fatality rate is primarily due to its metastatic nature, particularly spreading to the liver, brain, lungs, and bones [5]. Advances in early detection and treatment have significantly improved survival rates, but chemotherapy, a cornerstone of breast cancer management, introduces severe side effects that negatively impact patients' quality of life. Chemotherapy is administered as either neoadjuvant therapy (before surgery) or adjuvant therapy (after surgery), depending on tumor biology [6]. It is especially crucial in treating high-risk, early-stage breast cancer [7]. However, chemotherapy-related toxicities are substantial, leading to profound physical, psychological, and social challenges for patients [8]. Symptoms often appear in clusters, wherein multiple interrelated symptoms occur simultaneously and intensify each other´s effects [9]. These clusters are classified into low-burden and high-burden categories based on severity, with high-burden clusters being significantly more detrimental to well-being [10].

Patients exposed to chemotherapy often experience numerous distressing side effects, including fatigue, sleep disturbances, pain, anxiety, and depression, which collectively impair daily functioning and overall quality of life [11]. The presence of high-intensity symptom clusters has been associated with delayed recovery, increased healthcare costs, and reduced adherence to treatment [12]. Studies indicate that 72.3% of breast cancer patients experience high-burden symptom clusters, whereas only 27.7% report low-burden symptoms [4]. Therefore, understanding symptom clusters and their interconnected effects is essential for developing more effective management strategies [13]. While chemotherapy significantly increases survival rates, its cytotoxic nature leads to multiple distressing side effects [14,15]. However, the severity and persistence of these symptoms vary based on chemotherapy regimen, patient age, tumor characteristics, and individual health conditions [16]. Although many patients undergo multiple chemotherapy cycles, leading to cumulative toxic effects and prolonged symptom burden [17]. Researchers have identified key chemotherapy-related symptoms to include fatigue as the most frequently reported symptom, affecting nearly all chemotherapy patients [18]. It leads to muscle weakness, impaired daily activities, and emotional distress. More so, pain is claimed to affect a large proportion of patients due to chemotherapy-induced neuropathy, joint stiffness, and inflammation [19]. Poor sleep quality affects approximately 60% of chemotherapy patients and is often linked to anxiety, depression, and physical discomfort [20]. Emotional distress is exacerbated by chemotherapy side effects, affecting long-term well-being and treatment adherence [21]. Chemotherapy-induced toxicities frequently trigger inflammatory responses, further worsening symptom burden [22]. Studies have shown that chemotherapy patients often experience multiple symptoms simultaneously, creating a synergistic effect where symptoms exacerbate one another [23,24]. However, it has been argued that cancer progression and co-morbidity could have an impact on symptom burden and distress level experienced by breast cancer patients.

The concept of symptom clustering has gained significant attention in oncology, as symptoms rarely appear in isolation. Instead, they form patterns of co-occurrence, influencing disease progression, treatment tolerance, and overall functionality [25]. The most commonly reported clusters among breast cancer patients’ post-chemotherapy include: fatigue and pain often co-occur, leading to reduced physical activity, sleep deprivation, and emotional distress [26]. The literature claims that persistent fatigue is associated with muscle weakness, cognitive impairment, reduced motivation to engage in social activities, and emotional distress. These get heightened due to uncertainty about prognosis, body image changes, and financial burdens, and anxiety and depression are revealed to negatively impact treatment adherence, daily functionality, and social interactions [27]. Furthermore, some gastrointestinal disturbance clusters have been previously identified. Symptoms such as nausea, vomiting, diarrhea, and appetite loss occur frequently [28]. These symptoms affect nutritional intake, energy levels, and emotional well-being, further contributing to fatigue [25]. The cumulative impact of symptom clusters significantly reduces patients´ health-related quality of life (HRQoL), leading to decreased independence and increased psychological distress. Studies have reported that chemotherapy-induced symptoms worsen over time, especially after three cycles, resulting in a gradual decline in well-being [15].

Several factors influence the severity and persistence of symptom clusters, including demographic factors, such as age, socioeconomic status, and genetic predisposition, which affect symptom severity [29]. Tumor biology and chemotherapy regimen influenced more aggressive cancers and intensive chemotherapy protocols, resulting in higher toxicity and symptom burden. Likewise, patients with strong support networks report lower distress levels and better coping mechanisms [30]. The emerging concept of a symptom network offers a new framework for analyzing symptom interactions, helping healthcare providers identify central symptoms that should be prioritized for intervention. Given the negative impact of chemotherapy-induced symptoms on quality of life, a comprehensive, multidisciplinary approach is essential for symptom management. This includes: regular patient assessments to monitor symptoms and their progression [12]; personalized symptom management plans focusing on pain relief, psychological support, and physical rehabilitation [4]; non-pharmacological interventions such as exercise, mindfulness, and cognitive-behavioral therapy have shown effectiveness in managing symptoms [31]; and adjusting doses and incorporating supportive medications to reduce toxicity. Breast cancer treatment, particularly chemotherapy, significantly improves survival rates but also imposes a substantial symptom burden that deteriorates quality of life.

Research has shown that unmanaged chemotherapy-induced symptoms can result in functional decline, increased healthcare costs, and prolonged recovery times. A multifaceted, patient-centered approach that emphasizes early symptom identification, targeted interventions, and supportive care strategies is essential to mitigate the negative impact of chemotherapy. Thus, this study aims to answer the gap in knowledge in the following areas, such as evaluating the impact of sociodemographic factors on symptom burden and QoL, the influence of cancer stage on symptom burden and QoL, the relationship between chemotherapy and symptom burden, QoL, and psychological wellbeing and the effect of multidimensional support system in allaying the burden experienced by breast cancer patients. Therefore, the main objectives of this study were to assess the levels of symptom burden and the effect of symptom burden experienced by breast cancer patients after undergoing chemotherapy. Also, to examine the relationship between demographic factors and symptom burden and quality of life of breast cancer patients after undergoing chemotherapy.

## Methods

**Study design:** this study employed a descriptive cross-sectional survey design to examine the relationship between symptom burden and its effect on the quality of life of breast cancer patients after undergoing chemotherapy. The study population was drawn from two tertiary hospitals (Delta State University Teaching Hospital, Oghara, and Federal Medical Centre, Asaba) between September and December 2024.

**Setting, sampling, and respondents´ recruitment:** the study focused on patients who had completed chemotherapy within the past 12 weeks at the oncology outpatient clinic. A purposive sampling technique was applied based on the specific eligibility criteria. Of the 195 patients recorded from the oncology clinics in both centers, 143 patients were recruited using the Cochran formula of sample size estimation, but only 139 completed the study and were included in the final analysis. Respondents were selected based on inclusion and exclusion criteria to maintain data accuracy and relevance. Eligible respondents were required to be at least 18 years old, have completed chemotherapy for breast cancer within 12 weeks, experience chemotherapy-related symptoms, and voluntarily agree to participate. Exclusion criteria included cognitive impairments, language barriers, pre-existing medical conditions that could confound symptom reporting, concurrent treatments (e.g., radiotherapy, hormonal therapy, and surgery), enrollment in other clinical trials, or diagnoses of other cancers.

**Study measurement:** the research instrument comprised three sections. Section A collected demographic and clinical data, such as age, marital status, education, occupation, cancer stage, and post-chemotherapy duration. Section B assessed symptom prevalence and severity using the Memorial Symptom Assessment Scale (MSAS), which evaluates 32 symptoms on a 0-4 scale. Section C measured quality of life with the City of Hope Quality of Life Instrument, assessing four domains (physical, psychological, social, and spiritual) on a 0-10 scale. Both tools demonstrated strong reliability and validity in prior studies. The MSAS has been validated in previous research, demonstrating strong internal consistency with Cronbach´s alpha = 0.782 to 0.94 [32]. Similarly, the City of Hope Quality of Life Instrument has proven reliability (test-retest reliability = 0.89, internal consistency = 0.71 to 0.93).

**Variables:** the various variables were analyzed to identify associations with symptom burden and quality of life.

**Dependent variables:** these are the outcome of the influence of the independent variable. They include symptom burdens and the quality of life of breast cancer patients.

**Independent variables:** the factors such as age, gender, educational status, occupation, marital status, time since diagnosis of cancer, cancer stage, and length of chemotherapy treatment.

Descriptive statistics were used to analyze the study variables. Categorical variables (such as sociodemographic characteristics, clinical characteristics, and the presence of specific chemotherapy-related symptoms) were summarized using frequencies and percentages to describe their distribution in the sample. Continuous variables (such as total symptom burden scores and quality-of-life domain scores) were analyzed using means and standard deviations to determine average levels and variability. Ranking order was applied to identify the most severe and frequently experienced symptoms, as well as the most affected quality-of-life domains. These descriptive analyses addressed research questions 1 and 2, which focused on identifying the symptom burden and determining the domains of quality of life most affected among breast cancer patients after chemotherapy. Principal component analysis performed in R (version 4.3.1) was used to explore underlying dimensions within the symptom-burden and quality-of-life scales, identifying clusters of related symptoms and components of QoL impairment. This analysis addressed research question 3. Inferential statistics were used to examine the association between symptom burden and overall quality of life, addressing research question 4. Data suitability was confirmed using the Kaiser-Meyer-Olkin (KMO) test and Bartlett´s Test of Sphericity. Varimax rotation was applied, with eigenvalues >1.0 and factor loadings >0.4, including only symptoms occurring in at least 20% of respondents. Chi-square tests were used to examine associations between symptom burden, quality of life, and sociodemographic factors. Symptom burden was categorized based on frequency and severity scores, with statistical significance set at p < 0.05.

**Ethical approval and consent to participate:** ethical approval was obtained before data collection (HREC/PAN.2024/056.0663 and FMC/ASB/A81VOL. XII/454). Respondents were recruited during outpatient visits and provided with detailed study information. Informed consent was obtained, participation was voluntary, and withdrawal was permitted at any stage. Confidentiality was maintained, and data access was restricted to authorized researchers. The study posed no harm, and counseling resources were available for distressed respondents.

## Results

**Descriptive characteristics of the study respondents:** the respondents in the study had ages ranging from 21 years and older. The majority were in the 41-50 years age group (32.37%), followed by 51-60 years (28.77%) and 61 years and above (24.46%). A smaller proportion was aged 31-40 years (10.79%) and 21-30 years (3.59%). The age distribution suggests that middle-aged and older adults were more commonly affected by the condition under investigation, likely cancer. Regarding education, nearly half of the respondents (49.64%) had completed tertiary education, with 35.97% having secondary education and 14.38% having primary education. The occupational status showed that 32.37% were civil servants, 28.77% were housewives or unemployed, 25.17% were private sector employees, and 13.66% were retired. This indicates a diverse working background, though many respondents were currently not employed. Most respondents were married (64.74%), with others being widowed (17.26%), single (10.79%), or divorced (7.19%). The marital status suggests that a majority had familial or spousal support, which could be beneficial during illness and treatment.

The time since diagnosis varied: 28.77% were diagnosed within the last year, 25.17% had been diagnosed 1-2 years ago, 28.77% had a diagnosis for 3-5 years, and 17.26% had been living with the diagnosis for over five years. This indicates that many respondents had recent diagnoses. Cancer stages were predominantly stage III (35.97%) and stage II (32.37%), with stage IV representing 17.26% and stage I 14.38%. This suggests that many were diagnosed at moderate to advanced stages of cancer. In terms of chemotherapy treatment duration, the highest proportions had treatment lengths of 7-8 weeks (28.77%) and 5-6 weeks (25.17%). A smaller number had treatment durations of 3-4 weeks (14.38%), 9-10 weeks (17.26%), 1-2 weeks (7.19%), and 11-12 weeks (7.19%), reflecting a trend toward moderate chemotherapy regimens.

**Symptom burden experienced by breast cancer patients after undergoing chemotherapy treatment in Delta State, Nigeria:** respondents reported varying levels of symptom burden (Table 1), with 35.97% indicating moderate burden, 21.58% reporting mild burden, 21.58% severe burden, and 13.66% very severe burden. Only 7.19% reported no burden, highlighting that over 92% of respondents experienced some level of symptom distress. A Chi-square test showed no significant relationship between age, education, or occupation and symptom burden. However, there was a significant relationship between marital status and symptom burden (χ^2^= 19.674, p = 0.034), with married respondents reporting moderate symptom burden more frequently, while widowed respondents experienced higher levels of severe symptoms (Table 2). A significant relationship was also found between the duration since diagnosis and symptom burden (χ^2^= 11.149, p = 0.047), with those recently diagnosed (within a year) reporting higher severe and very severe burdens. Additionally, cancer stage and symptom burden were significantly correlated (χ^2^= 8.292, p = 0.007), with those in advanced stages (stage IV) reporting higher levels of severe symptoms.

**Table 1:** levels of symptom burden among breast cancer patients after undergoing chemotherapy treatment in two tertiary hospitals, Delta State, Nigeria (n=139) as from September to December 2024

Symptom burden level	Frequency (n=139)	Percentage (%)
No burden	10	7.19
Mild burden	30	21.58
Moderate burden	50	35.97
Severe burden	30	21.58
Very severe burden	19	13.66

**Table 2:** relationship among sociodemographic characteristics and symptom burdens experienced by breast cancer patients after undergoing chemotherapy treatment in two tertiary hospitals, Delta State, Nigeria (n=139) as from September to December 2024

Variables		No	Mild	Moderate	Severe	Very severe	χ^2^	P value
Age	21-30	1 (6.7%)	3 (20.0%)	5 (33.3%)	4 (26.7%)	2 (13.3%)	11.187	1.191
	31-40	2 (8.0%)	5 (20.0%)	8 (32.0%)	7 (28.0%)	3 (12.0%)		
	41-50	3 (5.5%)	15 (27.3%)	20 (36.4%)	10 (18.2%)	7 (12.7%)		
	51-60	2 (4.2%)	12 (25.0%)	18 (37.5%)	10 (20.8%)	6 (12.5%)		
	61 and above	2 (5.4%)	10 (27.0%)	12 (32.4%)	8 (21.6%)	5 (13.5%)		
Education	Primary	3 (9.1%)	7 (21.2%)	10 (30.3%)	8 (24.2%)	5 (15.2%)	7.428	0.303
	Secondary	4 (8.0%)	12 (24.0%)	18 (36.0%)	10 (20.0%)	6 (12.0%)		
	Tertiary	5 (8.3%)	13 (21.7%)	22 (36.7%)	12 (20.0%)	8 (13.3%)		
Occupation	Housewife	3 (7.5%)	8 (20.0%)	12 (30.0%)	10 (25.0%)	7 (17.5%)	11.612	0.721
	Private employee	2 (6.1%)	7 (21.2%)	10 (30.3%)	9 (27.3%)	5 (15.2%)		
	Civil servant	4 (8.3%)	10 (20.8%)	15 (31.2%)	12 (25.0%)	7 (14.6%)		
	Retired	1 (4.2%)	5 (20.8%)	8 (33.3%)	6 (25.0%)	4 (16.7%)		
Marital Status	Single	2 (9.5%)	5 (23.8%)	6 (28.6%)	5 (23.8%)	3 (14.3%)	19.674	0.034
	Married	7 (7.8%)	20 (22.2%)	35 (38.9%)	18 (20.0%)	10 (11.1%)		
	Divorced	1 (7.1%)	3 (21.4%)	5 (35.7%)	3 (21.4%)	2 (14.3%)		
	Widowed	2 (6.1%)	6 (18.2%)	10 (30.3%)	9 (27.3%)	6 (18.2%)		
Duration since diagnosis	< 1 year	3 (7.5%)	7 (17.5%)	12 (30.0%)	10 (25.0%)	8 (20.0%)	11.149	0.047
	1-2 years	2 (6.5%)	6 (19.4%)	10 (32.3%)	8 (25.8%)	5 (16.1%)		
	3-5 years	4 (8.0%)	12 (24.0%)	18 (36.0%)	10 (20.0%)	6 (12.0%)		
	>5 years	2 (6.7%)	6 (20.0%)	10 (33.3%)	7 (23.3%)	5 (16.7%)		
Cancer stage	Stage I	4 (12.5%)	8 (25.0%)	10 (31.2%)	6 (18.8%)	4 (12.5%)	8.292	0.007
	State II	5 (9.3%)	12 (22.2%)	20 (37.0%)	10 (18.5%)	7 (13.0%)		
	Stage III	2 (5.7%)	7 (20.0%)	12 (34.3%)	8 (22.9%)	6 (17.1%)		
	Stage IV	1 (3.7%)	5 (18.5%)	8 (29.6%)	6 (22.2%)	7 (25.9%)		
Length of chemotherapy	1-2 weeks	2 (10.0%)	4 (20.0%)	6 (30.0%)	5 (25.0%)	3 (15.0%)	7.732	0.790
	3-4 weeks	3 (8.8%)	7 (20.6%)	12 (35.3%)	8 (23.5%)	4 (11.8%)		
	5 to 6 weeks	4 (8.0%)	10 (20.0%)	18 (36.0%)	12 (24.0%)	6 (12.0%)		
	7 to 8 weeks	3 (7.1%)	8 (19.0%)	15 (35.7%)	10 (23.8%)	6 (14.3%)		
	9 to 10 weeks	2 (6.7%)	6 (20.0%)	10 (33.3%)	7 (23.3%)	5 (16.7%)		
	11 to 12 weeks	1 (4.5%)	5 (22.7%)	7 (31.8%)	5 (22.7%)	4 (18.2%)		

Note: the p-value of less than 0.05 is considered statistically significant and suggests a meaningful association between the sociodemographic variable and symptom burden within the study population

**Quality of life (QoL) experienced by breast cancer patients after undergoing chemotherapy treatment in Delta State, Nigeria:** QoL was assessed across four domains: physical, psychological, social, and spiritual well-being. Physical well-being was reported as low or very low by 49.7% of respondents, with 33.8% reporting moderate levels and only 16.5% reporting high or very high well-being. Psychological well-being was low or very low in 50.4% of respondents, with moderate well-being in 30.9% and high or very high well-being in 18.7%. Social well-being was low or very low for 55.3% of respondents, with moderate well-being in 29.5% and high or very high in 15.1%. Spiritual well-being was low or very low in 45.3%, moderate in 31.7%, and high or very high in 23.7%.

The overall QoL showed that 50.2% of respondents had low to very low quality of life, 31.5% reported moderate QoL, and 18.5% reported high or very high QoL. Respondents with no symptom burden reported the highest QoL, with 70% falling into the very high or high categories. As symptom burden increased, QoL declined; with respondents reporting severe symptom burdens having the poorest QoL outcomes (only 15.8% reported high or very high QoL). This relationship between symptom burden and QoL was statistically significant (χ^2^= 19.828, p = 0.0225) (Table 3).

**Table 3:** effect of symptom burden on quality of life experienced by breast cancer patients after undergoing chemotherapy treatment in two tertiary hospitals, Delta State, Nigeria (n=139) as from September to December 2024

Symptom burden level	Very high	High	Moderate	Low	Very low	Chi-square	DF	P-value
No burden	3 (30.0%)	4 (40.0%)	2 (20.0%)	1 (10.0%)	0 (0.0%)	19.828	16	0.0225
Mild	5 (16.7%)	10 (33.3%)	8 (26.7%)	5 (16.7%)	2 (6.7%)			
Moderate	4 (8.0%)	12 (24.0%)	18 (36.0%)	10 (20.0%)	6 (12.0%)			
Severe	2 (6.7%)	6 (20.0%)	12 (40.0%)	7 (23.3%)	3 (10.0%)			
Very severe	1 (5.3%)	2 (10.5%)	4 (21.1%)	8 (42.1%)	4 (21.1%)			

The p-value below 0.05 indicates a statistically significant association between symptom burden and quality of life within this study population

**Sociodemographic and clinical variables and QoL experienced by breast cancer patients after undergoing chemotherapy treatment in Delta State, Nigeria:** a Chi-square test of various sociodemographic and clinical variables against QoL revealed significant associations with marital status and duration since diagnosis (Table 4). Married respondents reported higher QoL scores (χ^2^= 16.871, p = 0.044), while singles and widowed respondents had lower scores. The duration since diagnosis also showed a significant relationship with QoL (χ^2^= 9.069, p = 0.017), with those diagnosed within the past 1-2 years reporting relatively higher QoL scores, possibly due to early-stage optimism or better treatment outcomes. In contrast, age, education, and chemotherapy duration did not significantly affect QoL, suggesting that factors like marital status and emotional support may be more influential in improving quality of life in this population.

**Table 4:** relationship between sociodemographic characteristics and quality of life experienced by breast cancer patients after undergoing chemotherapy treatment in two tertiary hospitals, Delta State, Nigeria (n=139) as from September to December 2024

Variables		Very high	High	Moderate	low	Very low	χ^2^	P value
Age	21-30	3 (8.6%)	7 (20.0%)	10 (28.6%)	9 (25.7%)	6 (17.1%)	14.319	0.991
	31-40	5 (10.0%)	10 (20.0%)	15 (30.0%)	12 (24.0%)	8 (16.0%)		
	41-50	7 (8.8%)	18 (22.5%)	25 (31.2%)	18 (22.5%)	12 (15.0%)		
	51-60	6 (8.7%)	15 (21.7%)	22 (31.9%)	16 (23.2%)	10 (14.5%)		
	61 and above	5 (8.6%)	12 (20.7%)	18 (31.0%)	14 (24.1%)	9 (15.5%)		
Education	Primary	6 (11.1%)	10 (18.5%)	18 (33.3%)	12 (22.2%)	8 (14.8%)	11.728	0.109
	Secondary	9 (10.6%)	18 (21.2%)	28 (32.9%)	18 (21.2%)	12 (14.1%)		
	Tertiary	11 (10.6%)	22 (21.2%)	35 (33.7%)	22 (21.2%)	14 (13.5%)		
Occupation	Housewife	5 (7.8%)	14 (21.9%)	20 (31.2%)	15 (23.4%)	10 (15.6%)	9.702	1.721
	Private employee	7 (11.7%)	12 (20.0%)	18 (30.0%)	14 (23.3%)	9 (15.0%)		
	Civil servant	8 (10.0%)	17 (21.2%)	25 (31.2%)	18 (22.5%)	12 (15.0%)		
	Retired	4 (8.9%)	9 (20.0%)	15 (33.3%)	10 (22.2%)	7 (15.6%)		
Marital status	Single	6 (11.1%)	10 (18.5%)	18 (33.3%)	12 (22.2%)	8 (14.8%)	16.871	0.044
	Married	12 (9.2%)	30 (23.1%)	40 (30.8%)	30 (23.1%)	18 (13.8%)		
	Divorced	4 (12.5%)	6 (18.8%)	10 (31.2%)	7 (21.9%)	5 (15.6%)		
	Widowed	5 (9.3%)	12 (22.2%)	18 (33.3%)	12 (22.2%)	7 (13.0%)		
Duration since diagnosis	< 1 year	7 (10.6%)	12 (18.2%)	22 (33.3%)	15 (22.7%)	10 (15.2%)	9.069	0.017
	1-2 years	6 (11.1%)	10 (18.5%)	18 (33.3%)	12 (22.2%)	8 (14.8%)		
	3-5 years	10 (11.4%)	18 (20.5%)	30 (34.1%)	18 (20.5%)	12 (13.6%)		
	>5 years	6 (10.7%)	10 (17.9%)	20 (35.7%)	12 (21.4%)	8 (14.3%)		
Cancer stage	Stage I	8 (13.1%)	12 (19.7%)	20 (32.8%)	12 (19.7%)	9 (14.8%)	11.512	0.097
	State II	12 (11.7%)	22 (21.4%)	35 (34.0%)	22 (21.4%)	12 (11.7%)		
	Stage III	7 (10.4%)	14 (20.9%)	22 (32.8%)	14 (20.9%)	10 (14.9%)		
	Stage IV	5 (9.3%)	10 (18.5%)	18 (33.3%)	12 (22.2%)	9 (16.7%)		
Length of chemotherapy	1-2 weeks	5 (10.9%)	10 (21.7%)	15 (32.6%)	10 (21.7%)	6 (13.0%)	5.933	0.673
	3-4 weeks	6 (10.3%)	12 (20.7%)	20 (34.5%)	12 (20.7%)	8 (13.8%)		
	5 to 6 weeks	9 (10.3%)	18 (20.7%)	30 (34.5%)	18 (20.7%)	12 (13.8%)		
	7 to 8 weeks	8 (11.0%)	15 (20.5%)	25 (34.2%)	15 (20.5%)	10 (13.7%)		
	9 to 10 weeks	7 (12.3%)	12 (21.1%)	18 (31.6%)	12 (21.1%)	8 (14.0%)		
	11 to 12 weeks	5 (12.2%)	9 (22.0%)	12 (29.3%)	9 (22.0%)	6 (14.6%)		

A p-value less than 0.05 signifies a statistically significant association between the sociodemographic variable and quality of life within this study population

## Discussion

Breast cancer remains a major global health challenge, with chemotherapy playing a central role in its treatment. However, chemotherapy is often associated with significant symptom burden, adversely affecting patients´ physical, emotional, social, and psychological well-being. This study, conducted among breast cancer patients in Delta State, Nigeria, highlights the prevalence, severity, and impact of chemotherapy-induced symptoms on quality of life. Understanding these effects is crucial for improving supportive care, enhancing treatment adherence, and optimizing patient well-being. Symptom burden refers to the cumulative impact of multiple distressing symptoms that affect daily functioning and overall quality of life. It encompasses symptom severity, duration, and the extent to which symptoms interfere with normal life activities. In this study, a significant proportion of respondents (36%) reported moderate symptom burden, while 35.3% experienced severe or very severe burden, highlighting the critical need for effective symptom management strategies. Chemotherapy-induced symptoms are often multifaceted, with physical, psychological, and cognitive symptoms interacting to exacerbate overall distress. Among the most commonly reported symptoms in this study were pain (80%) and fatigue (65%), both of which significantly impaired physical function. Psychological symptoms, including worry (65%), sadness (60%), and difficulty concentrating (49%), were also prevalent, emphasizing the emotional toll of chemotherapy. Sleep disturbances (60%) further exacerbated fatigue and emotional distress, creating a cycle of worsening symptoms. These findings align with global research indicating that symptom clusters often interact, compounding distress and diminishing quality of life [33].

The relationship between symptom burden and various sociodemographic factors was examined in this study, revealing that marital status, duration since diagnosis, and cancer stage significantly influenced symptom burden. However, age, education, employment, and chemotherapy duration were not found to have a significant impact. Despite this, younger patients reported higher levels of symptom burden, potentially due to concerns about fertility, social roles, and long-term health consequences [34]. Meanwhile, patients aged 41-60 reported a moderate burden, likely due to cumulative chemotherapy side effects and comorbidities [35]. While education level did not significantly correlate with symptom burden, it did influence symptom management. Patients with higher education levels were more proactive in seeking medical information and adhering to treatment regimens. Conversely, those with lower education levels struggled with health literacy, making it difficult to interpret medical advice and navigate the healthcare system. These findings align with studies emphasizing the role of health literacy in patient outcomes [36,37].

Employment status also played a crucial role, as unemployed and informally employed patients had limited access to supportive care services, including pain management, counseling, and nutritional support. Housewives, in particular, reported slightly higher levels of severe (25.0%) and very severe (17.5%) symptom burden compared to employed patients. Although the Chi-square test (χ^2^= 11.612, p = 0.721) found no significant association between employment and symptom burden, research suggests that financial stability and access to healthcare services play a significant role in symptom management [38]. More so, marital status was significantly associated with symptom burden (χ^2^= 19.674, p = 0.034), with married patients reporting lower severe burden (11.1%) compared to single (14.3%) and widowed (18.2%) patients. Widowed patients had the highest severe burden (27.3%), reinforcing research suggesting that lack of social support exacerbates symptom severity and impairs coping mechanisms [39]. Financial hardships were also found to contribute to increased stress and lower treatment adherence, leading to worsened symptoms [40].

Patients diagnosed within the past year experienced significantly higher distress and symptom burden, while long-term survivors exhibited better coping mechanisms (χ^2^= 11.149, p = 0.047). Newly diagnosed patients reported the highest severe (25.0%) and very severe (20.0%) burden, while long-term survivors had lower symptom distress. This suggests that symptom burden decreases over time with adaptation and access to supportive care. These findings are supported by research indicating that newly diagnosed patients experience heightened anxiety and uncertainty, contributing to greater symptom distress [39]. Cancer stage emerged as a critical determinant of symptom burden and quality of life. A significant proportion of respondents were diagnosed at advanced stages (stage III and IV), which was associated with higher symptom burden due to more intensive chemotherapy regimens and prolonged side effects such as chronic pain, fatigue, and gastrointestinal distress. Late-stage diagnosis also correlated with lower quality of life, as patients faced greater uncertainty and emotional distress. These findings align with global studies indicating that patients with advanced-stage breast cancer experience more intense symptoms and reduced quality of life [41].

The study found a strong association between cancer stage and symptom burden (χ^2^= 8.292, p = 0.007). Patients with stage IV breast cancer had the highest very severe burden (25.9%), compared to 12.5% in stage I. These results support prior research showing that symptom distress increases as the disease progresses due to tumor-related complications and more aggressive treatments [42]. Marital status influenced symptom burden, with married patients experiencing lower psychological distress due to spousal support. Conversely, widowed and divorced patients faced higher distress and symptom burden, as social isolation was linked to poorer treatment adherence and increased emotional distress. The absence of a caregiver also intensified physical discomfort, particularly for those needing assistance with daily activities. These findings align with research suggesting that spousal support enhances resilience, treatment adherence, and symptom relief [43,44]. Strengthening peer support networks and community-based interventions may help mitigate these effects. A clear inverse correlation was observed between symptom burden and quality of life. Patients experiencing no symptom burden reported the highest levels of well-being, with 30.0% indicating very high quality of life and 40.0% reporting high quality of life. However, as symptom burden increased, quality of life declined substantially. Patients with mild symptom burden showed a decline in well-being, with fewer individuals (16.7%) reporting very high quality of life and an increased proportion experiencing moderate to low well-being. This suggests that even mild symptoms, if left unmanaged, can significantly impact daily functioning and emotional well-being [45]. For those experiencing moderate symptom burdens, the impact on quality of life became more pronounced. Only 8.0% reported very high well-being, while 36.0% experienced moderate quality of life. Severe and very severe symptom burden further diminished well-being, with many patients reporting low or very low quality of life due to persistent pain, fatigue, gastrointestinal issues, and emotional distress [46]. Patients with very severe symptom burden had the poorest quality of life outcomes, with 42.1% experiencing low quality of life and 21.1% reporting very low well-being. These findings underscore the significant impact of unmanaged symptoms, as co-occurring symptoms severely limit physical activity, social engagement, and psychological stability [34].

The strong inverse association between symptom burden and quality of life highlights the urgent need for comprehensive symptom management strategies. The findings suggest that a multimodal approach, combining pharmacological and non-pharmacological interventions, is essential for alleviating symptom distress and improving patient outcomes. Evidence supports the use of exercise and physical therapy to reduce fatigue, cognitive-behavioral therapy (CBT) to address emotional distress and sleep disturbances, and nutritional support to enhance energy levels and overall well-being [47]. Additionally, an integrative care model addressing both physical and psychological aspects of symptom burden is crucial for long-term quality of life improvement [48,49]. Early psychological intervention, patient education, and peer support networks can help newly diagnosed patients develop effective coping strategies, ultimately reducing symptom burden and enhancing quality of life over time.

**Implications for clinical practice:** the findings of this study have significant implications for oncology care, particularly in the areas of symptom assessment, patient-centered care, and supportive interventions. Healthcare providers play a crucial role in ensuring that persons with breast cancer receive comprehensive symptom management throughout the chemotherapy journey. One key implication is the need to prioritize proactive symptom assessments by incorporating validated tools for evaluating fatigue, pain, and emotional distress. Given the strong correlation between symptom burden and QoL, nurses should adopt a patient-centered approach that emphasizes individualized symptom control. Healthcare providers should play an active role in facilitating psychosocial interventions. Given that psychological distress was a significant predictor of QoL, oncology nurses should incorporate mental health screenings, stress assessments, and referral pathways for counseling into routine care. Facilitating support groups can provide patients with a platform to share experiences, reduce emotional distress, and build resilience. More so, policy can be made to facilitate interdisciplinary collaboration, to strengthen work relationships among oncologists, psychologists, and palliative care specialists in designing ways to optimize treatment experiences and improve overall patient well-being.

**Limitations:** the study was conducted in selected tertiary hospitals, which may limit the generalizability of findings to other cancer care settings. The reliance on self-reported symptom burden and QoL assessments may introduce recall bias or social desirability bias. The study did not assess long-term changes in symptom burden and QoL, limiting the ability to determine whether improvements are sustained over time.

## Conclusion

This study examined symptom burden and its effect on the quality of life among a selected group of breast cancer patients who had undergone chemotherapy in Delta State, Nigeria. The findings showed that within this study population, sociodemographic and clinical characteristics influenced both symptom severity and overall QoL. Cancer stage, marital status, and time since diagnosis were particularly significant. Patients in advanced cancer stages reported more severe symptoms, while those diagnosed more recently experienced higher levels of severe and very severe symptoms. Marital status also played a role, with married respondents reporting lower symptom burden compared to widowed participants. Across the population studied, QoL was generally low, especially in the physical, psychological, and social domains. In contrast, factors such as age, educational attainment, and duration of chemotherapy did not show statistically significant associations with symptom burden or QoL. This suggests that clinical factors and psychosocial support may have a stronger influence on patient outcomes than demographic characteristics. The study underscores the need for holistic care that addresses physical, emotional, social, and spiritual needs for breast cancer patients undergoing chemotherapy in similar settings. These findings apply specifically to the study population and should not be generalized beyond this context. Further research in varied settings is recommended.

### What is known about this topic

- Cancer diagnosis and treatment often negatively impact patients' quality of life, affecting physical, psychological, social, and spiritual well-being;
- Symptom burden is a key predictor of quality of life, with higher symptom severity associated with lower QoL among cancer patients;
- Sociodemographic factors such as marital status and education have been shown in previous studies to influence coping mechanisms and quality of life outcomes in cancer populations.

### What this study adds

- This study identifies marital status and duration since diagnosis as significant predictors of overall quality of life among cancer patients;
- It demonstrates that symptom burden level is significantly associated with QoL, highlighting the need for better symptom management strategies;
- It also shows that variables such as age, education, occupation, and chemotherapy duration do not have a statistically significant impact on QoL, suggesting the influence of more personal or psychosocial factors.
